# Up against the wall - emergency double myocardial rupture repair

**DOI:** 10.1186/s13019-024-02531-2

**Published:** 2024-01-31

**Authors:** Rajeevlochan Ravi, Shubhra Sinha, Craig Dunlop, Jonathan Unsworth-White

**Affiliations:** 1 Cambridge University Hospitals NHS Foundation Trust, Cambridge, UK; 2 University Hospitals Plymouth NHS Trust, Plymouth, UK; 3 Birmingham Women’s and Children NHS Foundation Trust, Birmingham, UK

**Keywords:** Left ventricular free wall rupture, Interventricular septal rupture, Acute myocardial infarction, Double myocardial rupture, Free-wall rupture

## Abstract

**Background:**

Left ventricular free wall rupture (LVFWR) and interventricular septal rupture (VSR) are potentially catastrophic mechanical complications after acute myocardial infarction (AMI). When they occur together, “double myocardial rupture” (DMR), survival is unlikely. DMR is seen in only 0.3% of all AMIs. With or without surgical intervention, the odds are against the patient.

**Case presentation:**

A 57-year-old male self-referred to the emergency department of a remote hospital 5 days after first experiencing chest pain. Investigations in ED confirmed an inferior ST-segment elevation myocardial infarction (STEMI) complicated by DMR. Coronary angiography revealed a mid-course total occlusion of the right coronary artery (RCA). He was rapidly transferred to our regional cardiac surgical unit, arriving straight into the operating theatre, in cardiogenic shock. He was briefly conscious, before arresting prior to intubation and being massaged onto bypass. Not only did he survive the all-night operation, requiring a mitral valve replacement in the process, but he survived multiple postoperative complications to be eventually transferred on postoperative day 66, neurologically intact, to a peripheral unit to complete his rehabilitation. He was subsequently discharged home 88 days after the operation and was able to ambulate with a walking frame into his first postoperative follow-up clinic appointment.

**Conclusions:**

Our patient, against all odds, has survived DMR and multiple postoperative complications. We present the details of his case and the literature surrounding the condition. The patient’s mental fortitude and his supportive family played a significant role, along with excellent multidisciplinary team work, in assuring his survival.

**Supplementary Information:**

The online version contains supplementary material available at 10.1186/s13019-024-02531-2.

## Background

Left ventricular free wall rupture (LVFWR), and Inter-ventricular septal rupture (VSR) are major mechanical complications after acute myocardial infarction (AMI). The incidence of post-infarction LVFWR ranges from 2-6.2% [1] of AMI with an in-hospital mortality rate of 80% [2]. Post AMI VSR has an incidence of 0.25% [3]. Reperfusion interventions such as thrombolysis and percutaneous coronary intervention (PCI) have significantly reduced the incidence of these frequently catastrophic complications of AMI [3]. Without prompt surgical intervention, both VSR and LVFWR will usually prove fatal. We present the successful surgical management of such a patient.

## Case presentation

**Fig. 1:**
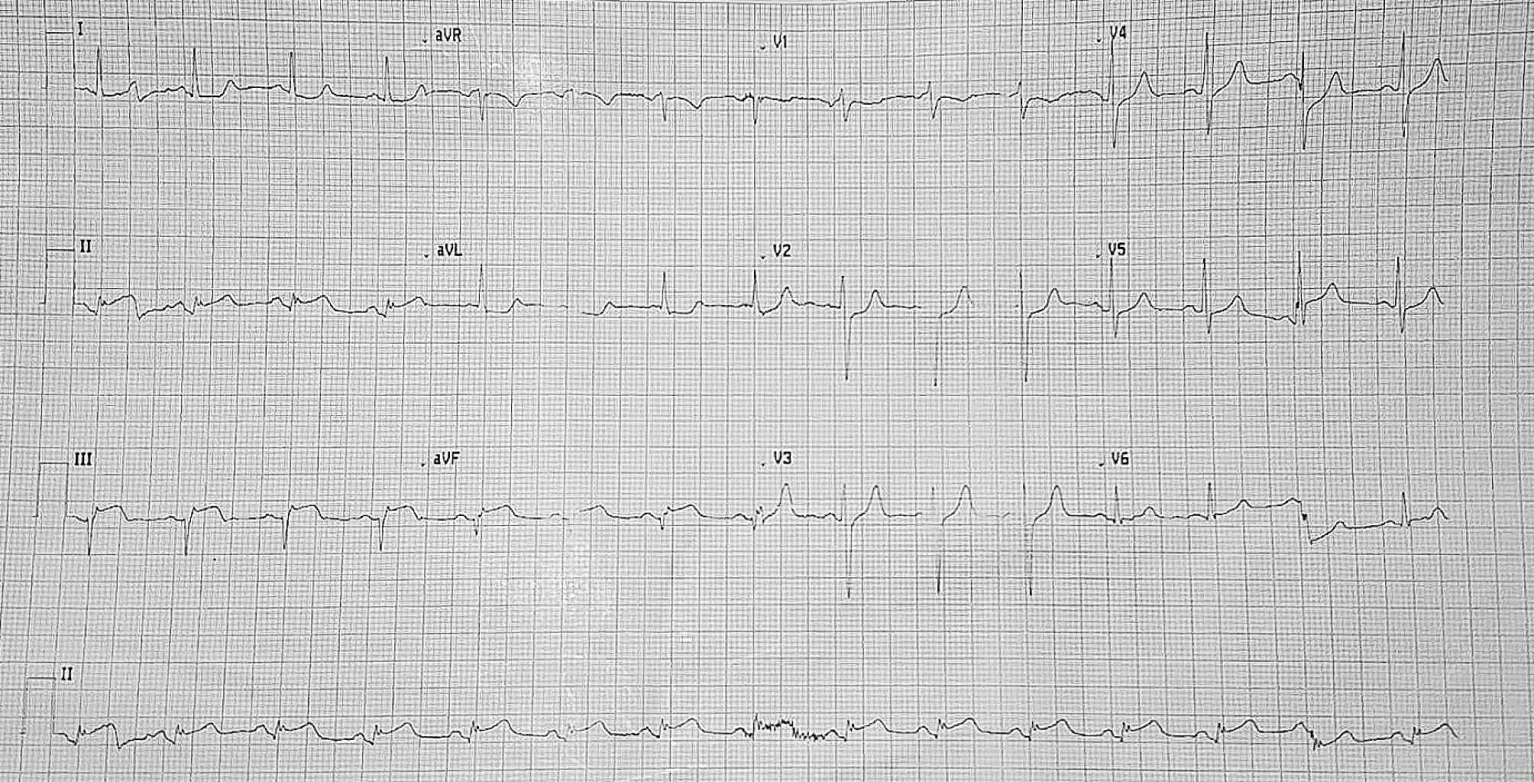
Illustrates the ECG which was taken pre-operatively and shows an established inferior infarction

A 57-year-old Caucasian male self-referred emergently to a peripheral hospital with sudden exacerbation of the chest pain he had experienced five days previously. This had been initially diagnosed as musculoskeletal pain by the primary care provider. The electrocardiogram (ECG) identified an inferior infarction as seen in Fig. 1.

**Fig. 2:**
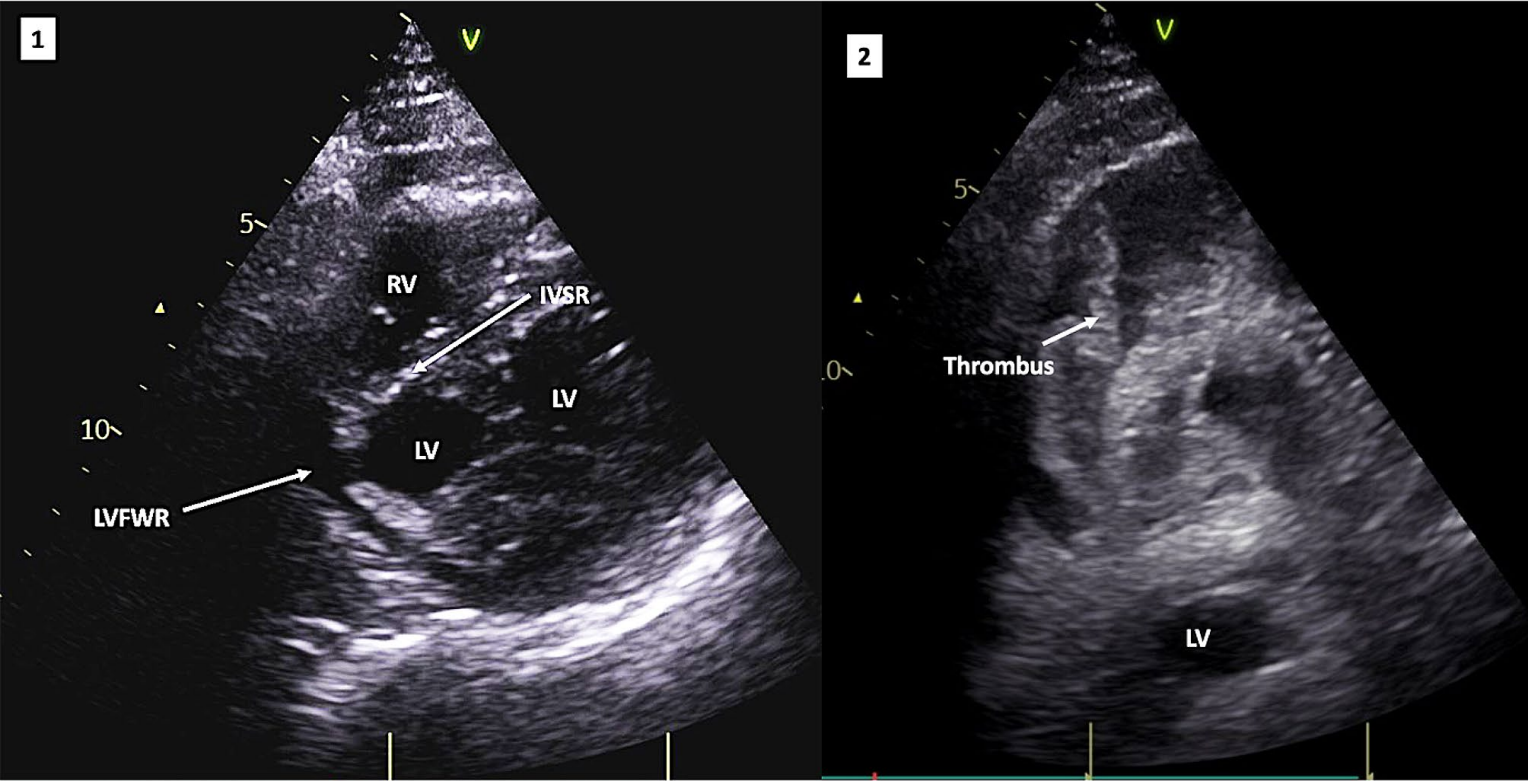
Left Image [**1**] is an echocardiographic image taken at the time of hospital admission. This is a parasternal short axis view. The image illustrates LVFWR, whilst moving images identified paradoxical movement. The areas of suspected VSR and LVFWR are identified by white arrows. Right Image [**2**] illustrates pericardial fluid and thrombus, supporting the diagnosis of LVFWR

**Fig. 3:**
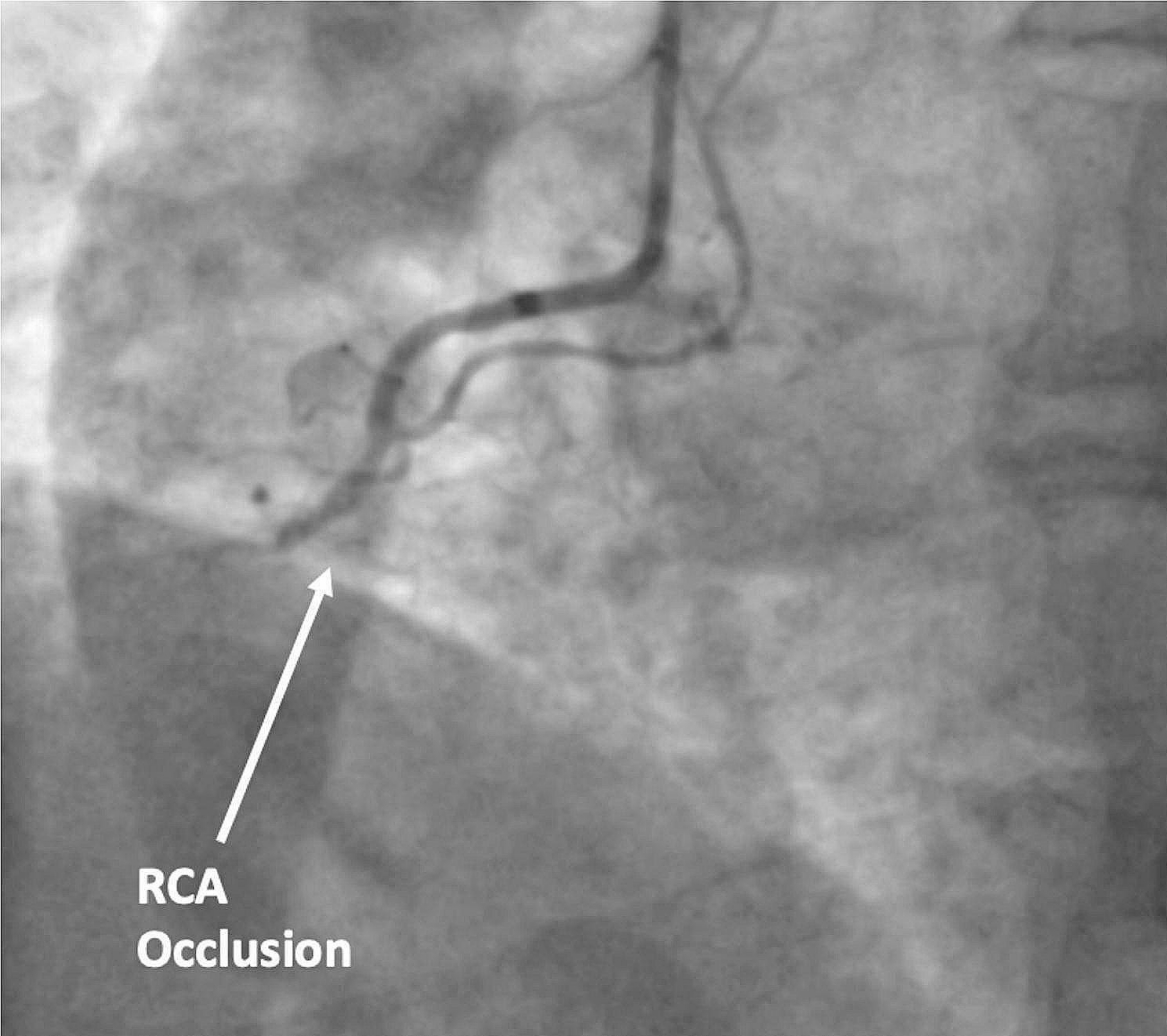
Still frame from our patient’s emergency coronary angiography shows RCA occlusion

Echocardiography in ED suggested LVFWR with pericardial thrombus and an inferior ventricular septal rupture (Fig. 2). Coronary angiography revealed a mid-course total occlusion of a dominant right coronary artery (RCA) and no left sided coronary disease (Fig. 3). He survived the 58-mile ambulance transfer to the regional cardiac surgical unit, arriving in cardiogenic shock but conscious.

## Operation & post-operative complications

### Operation

He was transferred straight to the operating theatre (OR) where he arrested prior to induction of anaesthesia. External compressions were commenced followed by a sternotomy and internal massage. Emergency right atrial/aortic cardio-pulmonary bypass (CPB) was established. The circuit was subsequently converted to bi-caval cannulation by advancing the two-stage cannula into the IVC and cutting in a separate Superior Vena Cava (SVC) angled cannula. A large volume of pericardial clot was removed. We were able to get transoesophageal echocardiography (TOE) Images as seen in the Video 1. The patient was cooled to 27 °C and the heart was arrested with 1 L of cold blood cardioplegia (St. Thomas’s solution), administered into the aortic root.

The apex of the heart was elevated with a pledgetted suture to access the inferior surface of the heart and the LVFWR. An inferior left ventriculotomy was performed through the infarct and free wall rupture, towards the base of the heart. After digital confirmation of the site of the VSR a large pericardial patch was sewn onto the LV side of the interventricular septum with 3 − 0 Prolene and brought out through the ventriculotomy. The ventriculotomy was closed using two long felt strips, sutured into place with multiple 1 − 0 Ethibond mattress sutures, oversewn with 1 − 0 Prolene, incorporating a third Teflon felt strip and abundant BioGlue. De-airing manoeuvres were performed, and the cross-clamp was removed. With the heart ejecting, TOE easily identified new torrential mitral regurgitation, which was not present pre-bypass. After arresting the heart once more, a posterior leaflet-sparing mitral valve replacement with a 29 mm ATS bi-leaflet mechanical valve was expeditiously performed via Sondergaard’s groove. The RCA was not grafted as the occlusion was mid-section and the patient’s clinical situation was concerning.

The heart was successfully weaned from bypass with pacing and moderately high doses of noradrenaline and adrenaline. The intraoperative TOE showed reasonable left ventricular function, moderate right ventricular function, no residual VSR and normal performance of the mechanical mitral valve. Inotropic support was soon reduced as systolic pressures exceeded 160mmHg at times. An intra-aortic balloon pump seemed superfluous. With haemostasis completed, the chest was closed.

However, it soon became evident that mediastinal blood loss precluded a CICU transfer, resulting in re-sternotomy. A new free wall rupture at the base of the heart adjacent to the IVC and ventricular closure was identified. The mitral prosthesis rendered cardiac displacement for attempted suture repair far too risky. By this stage, fresh frozen plasma, platelets, ocptaplex and cryoprecipitate had been infused. Against expectations, several minutes of digital pressure facilitated the temporary sealing of the hole. BioGlue and Surgicel were next deployed to tamponade the region, resulting in no further blood loss. The chest was soon re-closed, and the patient transferred to CICU in a haemodynamically stable condition. The total CPB and cross-clamp times were 191 min and 87 min respectively.

### Post-operative complications

Inotropic support was rapidly escalated over the first 48 h, until a Swan Ganz catheter was gingerly advanced past the VSR repair site and goal-directed fluid replacement facilitated inotropic weaning. Additional support included haemofiltration and, later, a tracheostomy. Remarkably, there was no neurological deficit whatsoever. A 1 L collection of haemoserous pericardial effusion was percutaneously drained on day 15 by which time Heparin-induced-thrombocytopenia (HIT) had been confirmed. He was finally free of renal support by day 25 and his tracheostomy was removed on day 41. Vasopressor-induced peripheral limb ischaemia caused dusky feet and toes, resulting in bilateral “mit” forefoot amputations on day 50. Several necrotic fingertips are still to demarcate.

The patient spent a total of 56 days in CICU before being transferred to ward-based care, and thence to a rehabilitation centre for continued mobility support on day 66. He was discharged home 88 days after the operation. He was able to ambulate into his first post operative clinic appointment.

## Discussion

Willian Harvey described LVFWR in 1647 at autopsy of a knight who had suffered severe chest pain [4]. Subsequently, VSR was described by Latham at an autopsy in 1847 [5], which led Brunn in 1923 [6] to make the first antemortem diagnosis of post-AMI VSR. The first surgical repair of VSR was reported by Cooley in 1957 [7]. It was 15 years later when Fitzgibbon reported the first successful surgical repair of the LVFWR [4].

**Fig. 4:**
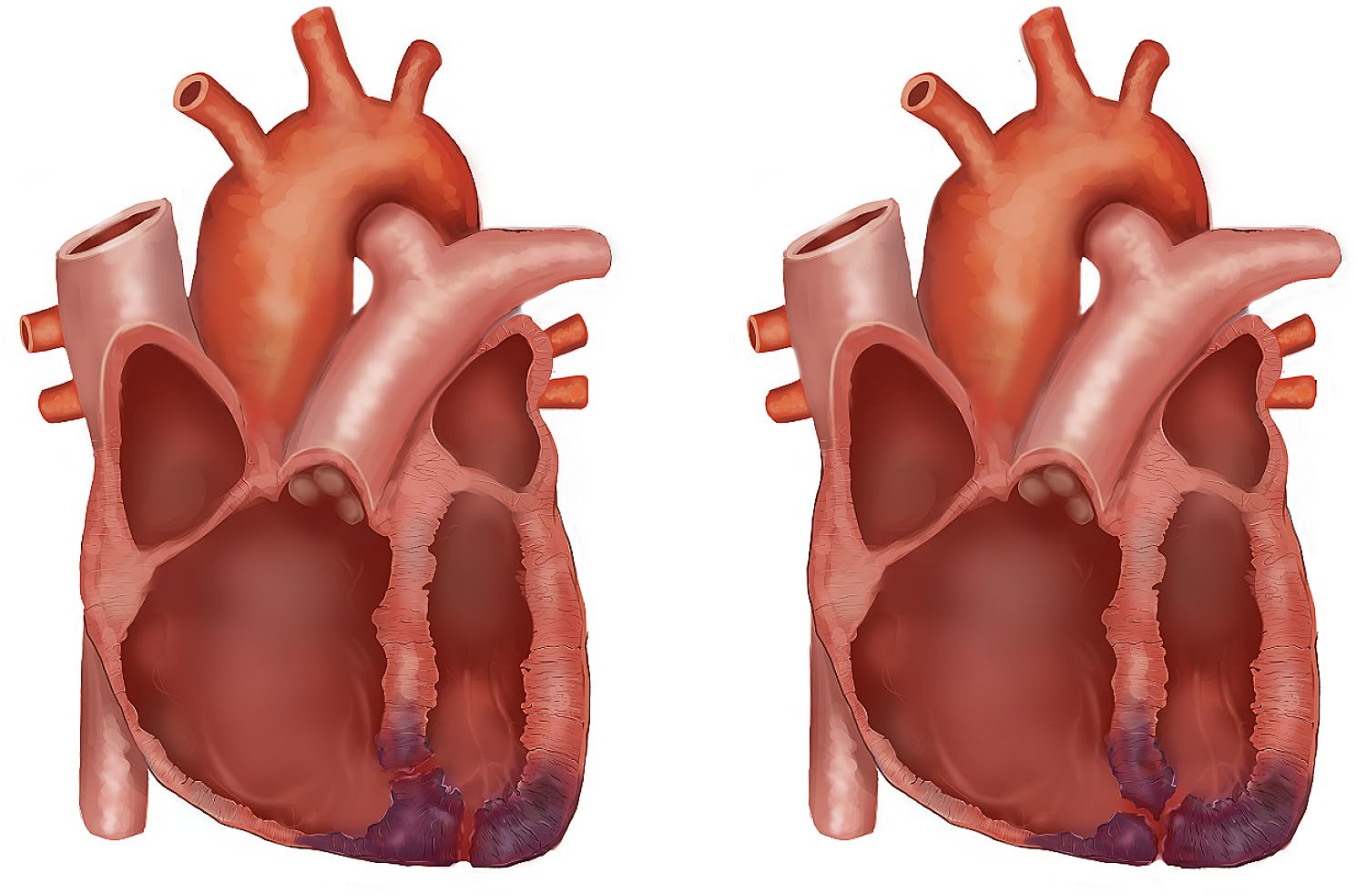
Adapted from Tanaka et al. (2003) illustrate the two types of DMR: true and junctional. The diagram shows the infarction area and how DMR could present [10]

The combination of LVFWR and VSR is termed “Double Myocardial Rupture (DMR)“ [8] and is a late presentation of AMI (1 to 14 days). Risk factors for developing DMR are patients over 65, relative freedom from coronary arterial disease, hypertension, the first AMI, continued activity (stuttering) AMI and delayed treatment [8]. Two forms of DMR, true and junctional, are described, as seen in Fig. 4 [9, 10]. The true form matches our patient (Video 1). The management and repair for junctional vs. true would be the same, using a single patch.

DMR is seen in 0.3% of all patients with AMI [9]. Patients who sustain any cardiac rupture have a high mortality rate and even after a successful surgical correction of DMR, the four month-survival rate post-operation remains high at 37.5% [9]. It should be mentioned delaying surgery is often appropriate. However, in this case the patient was tamponading and had arrested prior to induction of anaesthesia, fortunately in the operating theatre, so immediate surgery was imperative.

Cooley (1957) described using a polyvinyl sponge to plug VSR post-AMI and Daggett (1977) proposed infarctectomy and closure with a patch with a short suture line. However, both Cooley and Daggett’s techniques had drawbacks with sacrifice of healthy septal myocardium and a high incidence of post-operatively ventricular septal defects (VSD), mortality, and poor long-term survival [11]. The most widely used technique, as in our case is based on a procedure first attempted by David and colleagues in 1995, which used a pericardial patch, tissue glue and felt reinforcement. The technique retains healthy myocardium and has been found to have lower 30-day mortality and higher long-term survival [11, 12]. Nevertheless there is a 30% incidence of residual or recurrent shunt and, as in our case, a significant risk of compromising the mitral apparatus in inferior repairs [11].

There have been other more recent suggestions for VSR repair; Caimmi and colleagues (2010) proposed a double-patch technique via a right ventriculotomy in which two patches, supported by tissue glue, equally sandwich the infarcted septum between the left and right ventricles. Sugimoto and colleagues (2008) [13] have suggested the triple-patch technique in which the first pericardial patch closes the VSR, and the second patch is cut into two sufficient sizes, sutured to non-infarcted endocardium on the same side. The third patch is sutured to the non-infarcted endocardium on the ventricular free wall on the same side again. Finally, fibrin glue fills the cavity between the first patch and the pouch created with the second and third patches. Triple-patching is said to provide robustness with a solid barrier to prevent recurrent rupture and resistance to suture stress [14].

The use of tissue adhesive and surgical glues have facilitated suture-less techniques to treat LVFWR [15]. Digital pressure to achieve clot formation is followed by tissue glue directly onto the affected area with or without additional support from a cellulose product or pericardium to stabilize it [16]. This method was employed successfully in this patient when a new rupture was identified following initial chest closure. The subsequent bleed and the requirement to reopen was most likely due to the sutures cutting through the base of the heart where the tissues were infarcted and soft.

Our patient suffered a complicated postoperative recovery, surviving multiple organ failures. Organ failure has been associated with increased mortality, ranging from 12% (single organ failure) to more than 60% with double or triple organ failure [16, 17]. During his 56 days in ICU, he required prolonged ventilatory support, haemofiltration, and haematological support for heparin induced thrombocytopenia. Despite his arrest in tamponade, he escaped neurological compromise.

Time is of the essence in the management of AMI yet during the COVID-19 pandemic, there was a 38% decrease in STEMI presentations and a 48% decrease in AMI hospitalisations in the USA [1]. Others have confirmed delays in STEMI patients seeking medical attention during COVID-19 [19]. A 43% reduction in admission for AMI was noted in Italy [20]. Patient apprehension about contracting COVID-19 in hospital could have contributed to these findings. It is well known that mechanical complications post-AMI increase with pre-hospital delay and are associated with poorer outcomes [21]. Three case reports have shown that LVFWR has been rising throughout the COVID-19 pandemic as patients delay seeking care over the fear of contracting COVID-19 [1, 22, 23]. The NHS national cardiac audit programme (NCAP) 2022, showed 40% fewer patient were admitted with NSTEMI’s and there was a 25% reduction in STEMI admissions. Only 37% patients with STEMI received PCI, a key reperfusion intervention to reduce AMI complications [24].

It is appreciated that ST elevation is not always indicative of AMI. In this case it’s suggestive as the infarct occurred 5 days previous to the presenting with VSR and LVFWR. It would have been useful to take a thorough history and have serial biomarkers at that time to find out the exact timing of the AMI. Nevertheless, nearly a third (29.9%) of patients with AMI have been initially misdiagnosed prior to or on admission to hospital [25]. Common misdiagnoses include oesophageal reflux, gastritis and musculoskeletal pain, in both hospital and primary care. Early, accurate diagnosis is key to a successful outcome. Initial misdiagnosis (as in our patient) or patients simply failing to present for evaluation are common and contribute to the problem.

## Conclusions

Double myocardial rupture is usually a fatal mechanical complication of AMI. Our patient survived a double rupture against the odds, after arresting off tamponade prior to induction of anaesthesia and surviving multiple postoperative complications, The incidence of LVFWR and VSR had decreased over time due to rapid reperfusion strategies. However, delays in conjunction with the pandemic have increased the incidence of mechanical complications once more. Cardiac surgeons must therefore be prepared to tackle this rising problem. Although the operations are challenging and the postoperative issues demanding, good outcomes are still possible.

### Electronic supplementary material

Below is the link to the electronic supplementary material.

Supplementary Material 1

## Data Availability

If required, please contact the authors provided for data and materials.

## Abbreviations

LVFWR: Left ventricular free wall rupture
VSR: Interventricular septal rupture
AMI: Acute myocardial infarction
PCI: Percutaneous revascularisation
ECG: Electrocardiogram
STEMI: ST segment elevation myocardial infarction
CICU: Cardiac intensive care unit
RCA: Right coronary artery
CPB: Cardiopulmonary Bypass
IVC: Inferior vena cava
SVC: Superior vena cava
TOE: Transoesophageal echocardiogram
VSD: Ventricular septal defect
